# Digital communication tools used to promote healthy lifestyles: an overview of reviews from the BRIDGE project

**DOI:** 10.3389/fdgth.2026.1805124

**Published:** 2026-07-30

**Authors:** Khaoula El Kinany, Amira Abdelhamid, Arnold Benedict, Mohamed Aly, Osama Abdelkarim, Karima El Rhazi

**Affiliations:** 1 Laboratory of Health Sciences, Care and Health Technologies, Higher Institute of Nursing Professions and Health Techniques, Fez, Morocco; 2 Department of Epidemiology and Public Health, Faculty of Medicine and Pharmacy of Fez, Sidi Mohamed Ben Abdellah University, Fez, Morocco; 3 Department of New Media and Communication, School of Visual Arts and Creativity Management, ESLSCA University, Giza, Egypt; 4 Institute of Sports and Sports Science, Karlsruhe Institute of Technology, Karlsruhe, Germany; 5 Department of Sports Management, School of Business, ESLSCA University, Giza, Egypt; 6 Faculty of Sport Sciences, Assiut University, Assiut, Egypt; 7 Faculty of Medicine, Pharmacy and Dental Medicine, Department of Epidemiology, Clinical Research and Community Health, Hassan II University Hospital, Sidi Mohamed Ben Abdellah University, Fez, Morocco

**Keywords:** communication technologies, digital health tools, nutritional habits, physical activity, sedentary behavior, sleep quality

## Abstract

**Introduction:**

The widespread use of digital communication tools can contribute to promoting healthy behaviors within the population. The main aim of this study is to provide a comprehensive analysis of digital communication tools used to promote healthy lifestyles and to explore the specific techniques through which they facilitate behavior change.

**Material and methods:**

An overview of reviews was conducted by consulting PubMed, ScienceDirect and Cochrane and using the search syntax specific to each database. The selection criteria for included manuscripts focused on studies describing or evaluating digital health tools designed to promote healthy eating habits, physical activity, and sleep quality, and tools created to reduce sedentary behavior, whether implemented within research or intervention programs.

**Results:**

36 reviews were included in the final overview. Digital interventions to promote healthy lifestyles are most effective when they combine several behavior change techniques, including goal setting, self-monitoring, feedback, incentives, and social support. Among the most effective tools are mobile applications, wearable devices, web platforms, social networks, chatbots, and gamification systems. While gamification and social competition appear particularly effective with young people, personalized feedback, self-regulation, coaching, and problem-solving strategies are more beneficial for adults and older adults. Interventions offering a high level of personalization and interactivity generally yield the best results in terms of adopting healthy behaviors.

**Conclusion:**

The reviewed studies demonstrate that digital communication tools can effectively promote healthy and lasting behavioral changes and contribute to improved health outcomes, even remotely. This highlights the importance of tailoring digital interventions to age, motivation, and the cognitive abilities of users, while integrating appropriate behavior change techniques to optimize engagement and effectiveness.

## 1 Introduction

The 2030 Agenda for Sustainable Development recognizes noncommunicable diseases (NCDs) as a major global challenge for sustainable development that impact public health (1). NCDs, such as cardiovascular diseases, diabetes mellitus, hypertension, dyslipidemia, cancer, musculoskeletal disorders, depression, and cognitive impairment, are associated with prolonged sedentary time and physical inactivity (2). Also, NCDs are linked to inadequate sleep and poor nutrition (3).

Recently the rapid emergence and expansion of digital health communication tools have changed the behavior of many individuals towards healthier lifestyle (4). These digital health interventions include mobile applications, wearable devices, social media platforms, web-based programs, SMS interventions, chatbots, and AI-powered tools (5, 6).

Feedback, reminders, goal setting, social interaction, and gamification in digital communication tools can improve patient adherence to healthy behaviors (7). These tools encourage positive behavior change and improve individuals’ overall well-being through enhancing self-monitoring, providing motivation and reminders, showing personalized feedback, and sustaining long-term engagement (8, 9).

To promote healthier lifestyle, digital tools integrate established theoretical foundations of behavior change to optimize their effectiveness. Models such as the transtheoretical model (TTM), social cognitive theory (SCT), the COM-B model, and self-determination theory (SDT) offer frameworks that guide the design of digital features (10). For example, digital interventions often use behavior change techniques (BCTs) like goal setting and feedback to prompt action (11). This integration moves digital health beyond simple information delivery to theoretically grounded interventions that support users through various stages of change.

Despite promising advancements, the current research evidence regarding digital tools for health behavior change is fragmented across different lifestyle domains, such as physical activity (PA), diet, sleep quality, and sedentary behavior (SB) (12). Moreover, the absence of a unified approach highlights a significant need for a comprehensive synthesis of existing knowledge across diverse technologies and behavioral targets to guide future intervention designs.

Finally, the main aim of this synthesis is to comprehensively compile data from systematic reviews on the use of digital communication tools to promote healthy behaviors. More specifically, it aims to examine the effectiveness of various digital communication tools, including mobile applications, text messaging, social media platforms, web programs, wearable technologies, and other digital health interventions, in promoting behavioral changes related to PA, healthy eating, reduced SB, and sleep. Furthermore, this synthesis seeks to identify and analyze the BCTs embedded in these interventions, exploring the mechanisms by which digital communication tools influence health behavior. This paper synthesizes current evidence and identifies research gaps across various lifestyle domains to enhance the design of future digital interventions.

## 2 Material and methods

### 2.1 Search strategy

A comprehensive literature search was conducted in PubMed, ScienceDirect, and Cochrane. The search strategy was developed using three key concept groups: (1) digital health interventions, (2) healthy lifestyle behaviors, and (3) behavior change techniques (BCTs). Searches were restricted to systematic review studies published between January 2020 and December 2025. To ensure transparency and reproducibility, the complete database-specific search strategies, including the exact Boolean search strings employed in PubMed, ScienceDirect, and the Cochrane library, have been included in the Supplementary Table S1 (Table 1).

**Table 1:** General characteristics of included studies.

Study	Year	Study population	Study design	Sample size	Behavior targeted
Alley et al. (30)	2024	Older adults (≥60 years)	Umbrella review and meta-meta-analysis	28,198 participants	Promoting PA
Beck Silva et al. (36)	2024	Adolescents	Systematic review and meta-analysis	9,603 participants	Healthy eating
Bi et al. (14)	2024	College students	Systematic review and meta-analysis.	569 participants (ranged from 34 to 187 participants)	Promoting PA
Buja et al. (15)	2024	College students	Systematic review.	2,579 university students	Promoting PA or reducing SB
Chatterjee et al. (8)	2021	Children, adolescents, and adults	Systematic review	NR	Healthy lifestyle management (regular PA, healthy habits, and proper dietary patterns)
Chen et al. (34)	2025	Older adults	Systematic review and meta-analysis	Varied from 8 to 160 participants	SB (sitting time)
Curtin et al. (42)	2025	Adults	Systematic review and meta-analysis	23,000 participants	Healthy and sustainable diet behaviors.
D'Amore et al. (16)	2022	Older adults	Systematic review and meta-analysis	3,455 participants	Promoting PA
Daniels et al. (31)	2025	Adults (≥65 years)	Systematic review and meta-analysis.	3,055 participants	Promoting PA
Goodyear et al. (17)	2021	Young people and adults	Systematic review	NR	Promoting PA and dietary behaviors
Grotto et al. (46)	2024	Older adults	Scoping review	147 participants	Sleep quality, insomnia management
He et al. (18)	2021	Children and adolescents	Systematic review and meta-analysis	558 participants	Promoting PA
He et al. (33)	2024	Adults	Network meta-analysis	2,957 participants	Reduction of sedentary time and prolonged sitting
Laranjo et al. (19)	2021	Adults	Systematic review, meta-analysis, and meta-regression	7,454 participants	Promoting PA
Leppe-Zamora et al. (32)	2025	Office workers (≥18 years)	Systematic review and meta-analysis	1,164 participants	Reduce sedentary behavior (SB), increase in breaks from sitting
Lu et al. (47)	2025	College students and young adults	Systematic review and meta-analysis	5,251 Participants	Sleep quality, sleep parameters, and insomnia severity.
Melo et al. (43)	2025	Adolescents	Systematic review	31,971 participants (ranged from 29 to 7,890)	Promoting healthy dietary behaviors
Mersha et al. (20)	2024	Adults	Systematic review	NR	PA, dietary behaviors, weight management, smoking cessation, and other health-related lifestyle behaviors
Paramastri et al. (44)	2020	Adolescents and adults	Systematic review	NR	Dietary intake, and PA
Parés-Salomón et al. (35)	2024	Office employees in workplace settings	Systematic review and meta-analysis	3,529 participants	Reduce SB (sitting time)
Peng et al. (21)	2022	College students	Systematic review and meta-analysis of RCTs	8,333 participants	Promoting PA or reducing SB
Petkovic et al. (29)	2021	Adults	Systematic review	871,378 participants	Health behavior change (PA, body function outcomes, psychological health outcomes, and well-being)
Salamanca-Sanabria et al. (48)	2025	Adolescents	Systematic review and meta-analysis of RCTs	13,296 participants	Sleep behavior
Scarry et al. (45)	2022	Adults	Systematic review	1,638 Participants (ranged from 27 to 732 participants)	Diet quality improvement, and healthy eating behaviors
Schaafsma et al. (37)	2024	Adolescents	Systematic review	Ranged from 15 to 988 adolescents	Dietary intake
Shaluhiyah et al. (38)	2025	Mixed populations	Systematic scoping review	NR	Healthy behaviors for non-communicable disease prevention (PA, diet, tobacco-related behaviors)
Sina et al. (39)	2022	Children and adolescents	Systematic review	Ranged from 11 to 54603 participants	Diet
Talens et al. (40)	2025	Children and adolescents	Systematic review of RCTs	NR	Dietary behaviors, PA, and other lifestyle-related behaviors.
Tong et al. (28)	2021	Adolescents and adults	Systematic review, meta-analysis, and meta-regression	77,243 participants	Lifestyle behavior change (PA, and diet)
Tong et al. (2024) (22)	2024	Adults and adolescents in MENA	Systematic review and meta-analysis.	27 articles describing 22 interventions (*n* = 10) and 4 nonexperimental studies (*n* = 6,141)	Promoting PA or reducing SB
Wang et al. (24)	2025	Children and adolescents	Systematic review and meta-analysis.	7,472 participants	Promoting PA or reducing SB
Wang et al. (25)	2025	Adolescents and adults	Systematic review and meta-analysis of RCTs	2,446 participants and 240 parent-child dyads	Promoting PA, EH, and SB reduction
Wang et al. (23)	2024	Children and adolescents	Systematic review and meta-analysis.	5,643 participants	Increase PA and fitness
Watson-Mackie et al. (26)	2024	Young women	Systematic review	10,233 participants	Promoting PA and physical literacy
Xu et al. (27)	2022	Children, adolescents, and adults	Systematic review	9,977 participants (ranged from 7 to 3,637 participants)	Promoting PA
Zarnowiecki et al. (41)	2020	Parents of children	Review	NR	Children's nutrition through parental influence

### 2.2 Selection process

To ensure that the collection of studies is relevant to the research question, specific inclusion and exclusion criteria were applied. The inclusion criteria focused on reviews describing or evaluating communication technologies and digital health tools designed to promote healthy eating habits, PA, and sleep quality, whether implemented in research or intervention programs. Records that did not address digital health interventions, healthy lifestyle behaviors, or BCTs, as well as non-review articles, conference abstracts, editorials, protocols, and studies published outside the predefined time frame, were excluded.

Subsequently, duplicates were removed, and the titles and abstracts of the selected articles were independently reviewed by two reviewers (KE and AA) to assess their relevance and compliance with predefined inclusion criteria. Of the 863 articles initially identified, 36 were deemed relevant to the use of digital health tools for health promotion. The same reviewers then conducted a full-text review of the selected studies to confirm their eligibility for inclusion in this review. Any disagreement regarding the inclusion or exclusion of a study was discussed at each stage of the selection process until a consensus was reached. If a consensus could not be reached, a third reviewer (AB) made the final decision.

### 2.3 Data collection

The three reviewers extracted relevant data from each included study, encompassing the first author’s name, year of publication, study population, study design, sample size, targeted health behavior, digital communication tool, degree of personalization and interactivity, BCTs employed, as well as the main findings and reported outcomes.

Following the thematic analysis of the retrieved studies, the selected articles were classified into four principal areas of investigation: 1. PA interventions delivered through digital tools mainly mHealth app-based interventions; 2. reduction of SB delivered through digital tools (mainly computer prompts, digital media, and wearables); 3. sleep quality described and controlled by mHealth applications; and 4. nutrition controlled by digital health applications and social media.

### 2.4 Quality assessment

The methodological quality of the included systematic reviews was assessed using the JBI Critical Appraisal Checklist for Systematic Reviews and Research Syntheses (11 items) (13). Two reviewers conducted the quality assessment independently. Discrepancies were resolved through discussion and consensus.

## 3 Results

**Figure 1:**
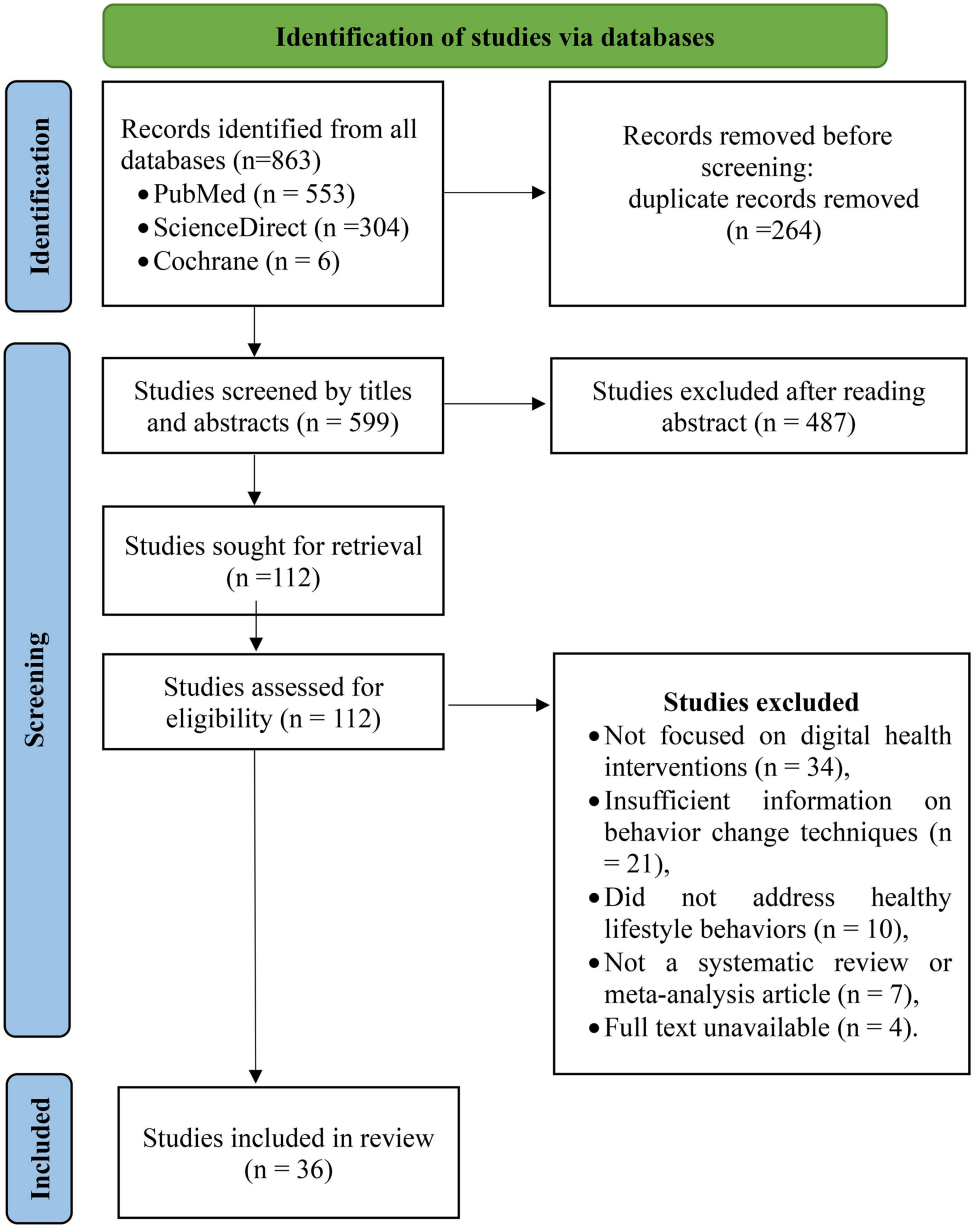
Preferred reporting items for systematic review and meta-analysis PRISMA 2020 flow diagram showing process of study selection.

The full texts of 112 articles were subsequently assessed for eligibility. Of these, 76 articles were excluded for the following reasons: not focused on digital health interventions (*n* = 34), insufficient information on BCTs (*n* = 21), did not address healthy lifestyle behaviors (*n* = 10), not a systematic review article (*n* = 7), full text unavailable (*n* = 4), Ultimately, 36 reviews met all inclusion criteria were included in the final overview. The complete study selection process is presented in the PRISMA flow diagram (Figure 1).

### 3.1 Physical activity (PA)

Evidence consistently demonstrates the effectiveness of digital communication tools in promoting PA among diverse populations, including children, adolescents, students, adults, and older adults (8, 14–31). Among children and adolescents, interventions based on gamification, exercise games, connected games, mobile applications, virtual reality, and social media platforms have significantly increased participation in moderate-to-vigorous physical activity (MVPA) and overall activity levels (17, 24, 26). Among college students, digital health interventions delivered via mobile applications, websites, wearable devices, social media, SMS and emails have significantly improved PA, including the number of daily steps (14, 21). Among adults, smartphone apps and activity trackers have increased PA by approximately 1,850 steps per day, while chatbot interventions have improved exercise participation and habit formation through ongoing guidance and support (19, 25). Among older people, smart technologies, including wearables, telehealth systems, video conferencing and mobile applications, have proven to be as effective as traditional face-to-face interventions in increasing PA and improving physical function (16).

The effectiveness of digital interventions aimed at promoting PA appears to be strongly linked to the integration of multiple BCTs, although the specific techniques employed vary according to the target population characteristics (8, 14–31). In children and adolescents, interventions were based primarily on gamification and strategies promoting social engagement, incorporating behavioral techniques, such as rewards and incentives, social comparison, social support, feedback and follow-up, goal setting, behavioral practice and reminders (17, 23, 24, 26). Among college students and young adults, digital PA interventions relied primarily on self-regulation strategies. Common BCTs included goal setting and planning, self-monitoring, feedback, social support, incentives and reminders, and behavioral regulation. Self-monitoring, particularly when combined with personalized feedback and clear activity goals, was identified as a key factor in the effectiveness of interventions, helping users track their progress, adapt their behaviors, and significantly increase their PA levels and daily step count (14, 21). In adults, digital PA interventions have primarily focused on self-regulation, personalization, and behavioral feedback (19, 25). Commonly used BCTs have included goal setting, action planning, self-monitoring, feedback, incentives, social support, behavioral regulation, and problem-solving. Personalized approaches, including chatbot interventions, have helped users overcome barriers to PA and develop tailored strategies. Self-monitoring and feedback have been shown to be among the most effective techniques for improving PA outcomes in adults (19, 25). For older adults, digital PA interventions focused more on ongoing monitoring, guidance, and support. Frequently used BCTs included goal setting and planning, self-assessment, feedback, educational support, social support, incentives and reminders, problem-solving, and behavior regulation. These interventions often offered personalized exercise recommendations, guidance, progress tracking, and reminders, with evidence suggesting that older adults particularly benefit from structured feedback, professional guidance, and clear instructions (16, 30, 31).

### 3.2 Sedentary behavior (SB)

Digital interventions targeting SB have primarily been evaluated among children, students, healthy adults, and older adults (21, 22, 24, 32–35). Mobile health (mHealth) interventions using smartphone apps, wearable activity trackers, accelerometers, and digital monitoring systems have been shown to significantly reduce sitting time among older adults, with reductions reaching 59 min per day (34). Similarly, wearable-device interventions among healthy adults effectively reduced total sedentary time and prolonged sitting episodes, particularly when combined with reminders and feedback mechanisms (33). Gamified interventions among children and adolescents demonstrated promising but less consistent effects on SB reduction (24). In contrast, eHealth interventions among college students successfully increased PA but did not consistently reduce SB (21). Leppe-Zamora et al. (32), and Parés-Salomón et al. (35), demonstrated the effectiveness of digital interventions in reducing SB and sitting time among office workers, with computerized interventions (32) and multicomponent strategies (35) integrating wearable devices, mobile applications, interactive dashboards, and personalized messages significantly reducing time spent sitting, the latter by an average of 29.9 min per 8-hour workday (35).

For SB interventions, the BCTs most frequently associated with effectiveness were feedback and monitoring, self-monitoring of behavior, prompts and cues, goals and planning, and behavioral regulation, although their implementation varied across populations and technologies (21, 22, 24, 32–35). In children, adolescents, and students, digital interventions incorporated rewards and incentives, social comparison, feedback and follow-up, social support, reminders and cues, goal setting, and self-monitoring; however, while these BCTs consistently increased levels of PA, their effectiveness in reducing SB was generally less consistent (21, 24). Among older adults, the mHealth interventions primarily incorporated self-monitoring, personalized feedback, goal setting, action planning, and prompts to interrupt prolonged sitting, which contributed to significant reductions in sedentary time (34). Similarly, the wearable-based interventions relied heavily on real-time feedback, activity monitoring, sedentary alerts, goal setting, and behavioral regulation, with reminder-based interventions showing the greatest effectiveness (33). In occupational settings, a meta-analysis demonstrated the effectiveness of computerized prompt interventions that primarily used prompts and cues and behavioral regulation (32), whereas the multicomponent interventions combined self-monitoring, personalized feedback, tailored messaging, goal setting, and continuous monitoring through wearable devices and mobile applications, resulting in significant reductions in workplace sitting time (35).

### 3.3 Dietary habits

Digital interventions targeting dietary behaviors have been extensively studied among children, adolescents, college students, parents, and the general population (8, 17, 20, 36–45). Among children and adolescents, smartphone apps, online programs, websites, social media, and SMS interventions have generally improved healthy eating behaviors, including increasing fruit and vegetable consumption, making healthier food choices, and reducing consumption of unhealthy foods (37, 40, 43). Computer-based nutrition interventions demonstrated modest improvements in dietary outcomes among adolescents, particularly reducing fat consumption (36). Interventions directed at parents to improve children's nutrition used websites, mobile applications, SMS, and email communication, resulting in improvements in parental nutrition knowledge, feeding practices, and children's dietary behaviors (41). A meta-analysis of mobile application-based interventions in the general population revealed significant improvements in eating behaviors, including increased fruit and vegetable consumption, reduced consumption of energy-rich foods, and better adherence to sustainable diets (42). Broader digital lifestyle interventions delivered through social media and internet-based group programs also reported positive effects on healthy eating and weight-related outcomes (17, 20, 40).

The most frequently identified BCT clusters were knowledge shaping, goals and planning, feedback and monitoring, self-monitoring, prompts and cues, and social support (8, 17, 20, 36–45). Across all the reviewed studies, digital dietary interventions generally integrated several BCTs. Beck Silva et al., found that computerized interventions emphasized nutrition education (knowledge structuring), monitoring and feedback, and goal setting to improve adolescents’ eating behaviors (36). Similarly, Schaafsma et al., reported frequent use of behavioral self-monitoring through food diaries, personalized feedback, goal setting and planning, reminders and incentives (e.g., notifications), and rewards and incentives offered by smartphone app and gamified app features (37). Focusing more specifically on engagement and adherence, Melo et al., identified follow-up and feedback, self-monitoring, goal setting, knowledge structuring, reminders and prompts, social support, and rewards and incentives as the most frequently used BCT groups (43). Overall, the dominant BCT groups underpinning the effectiveness of digital dietary interventions emerged as feedback and follow-up, self-monitoring, goal setting and planning, knowledge structuring, prompts and cues, and rewards and incentives (36, 37, 43).

### 3.4 Sleep outcomes

Sleep was the least frequently investigated lifestyle domain but showed promising results (46–48). In adolescents, digital health interventions delivered through mobile applications, web platforms, digital cognitive behavioral therapy for insomnia (dCBT-I), wearable systems, and online sleep management tools have shown to significantly improve sleep duration, sleep quality, sleep hygiene behaviors, insomnia symptoms, and sleep efficiency (48). Among college students and young adults, digital sleep interventions delivered through web platforms and mobile applications, including cognitive behavioral therapy for insomnia (CBT-I), mindfulness programs, and sleep education interventions, have shown to significantly improve sleep quality, insomnia severity, and sleep efficiency (47). Mobile health technologies disseminated through mobile applications, SMS reminder systems and passive sleep monitoring tools have shown modest and variable improvements in sleep quality among the elderly, although the available evidence remains limited (46). Their review identified relatively few eligible studies, highlighting a lack of evidence for this population (46).

The most important BCT clusters associated with improved sleep outcomes were feedback and monitoring, self-monitoring of behavior, goals and planning, knowledge shaping, prompts and cues, and behavioral regulation (46–48). Among adolescents, the effectiveness of digital interventions was primarily attributed to the integration of several BCTs, including feedback and monitoring, self-monitoring of behavior, goals and planning, knowledge shaping, prompts and cues, behavioral regulation, problem solving, and action planning (48). Sleep diaries, personalized feedback, educational modules, and goal-setting strategies were among the most frequently implemented components, with multicomponent interventions generally outperforming information-only approaches (48). In college students and young adults, the most common BCTs included self-monitoring of behavior, feedback and monitoring, goals and planning, cognitive restructuring, rewards and incentives, and stress management strategies (47). These components helped participants identify unsuitable sleep habits, track their progress, set sleep-related goals and develop healthier sleep routines, thus contributing to better sleep quality (47). In older adults, the most frequently reported techniques included knowledge shaping (education), self-monitoring of behavior, prompts and cues (e.g., SMS reminders), and occasionally goals and planning (46). Unlike interventions designed for younger populations, less emphasis was placed on feedback, cognitive-behavioral strategies, or interactive problem-solving, which may partly explain the smaller and more inconsistent improvements observed in sleep outcomes among older adults (46).

## 4 Discussion

This overview of reviews describes and analyzes digital communication tools used to promote healthy lifestyles based on specific techniques by which they facilitate behavior change. Our results showed that communication tools led to favorable lifestyle changes, particularly regarding eating habits, sleep quality, and PA. One of the main conclusions of our study is that the behaviors studied (sleep quality, nutrition, PA, and SB) were not addressed together in a way that explained the interaction between them. The most combination studied were PA and SB.

Digital communication tools have proven their effectiveness in promoting PA in various contexts (8, 14–31). Their effectiveness is linked to several factors, including the age group (children, young and old), the degree of personalization, the interactivity, and the BCTs clusters used (8, 14–31). Overall, despite age-related differences, several groups of BCTs have been shown to be consistent throughout life as key factors in intervention effectiveness, including goal setting and planning, feedback and follow-up, self-monitoring of behavior, incentives and cues, and social support (8, 14–28, 30, 31). The BCTs suggest that digital interventions achieve the best PA outcomes when these techniques are combined and tailored to the developmental and motivational characteristics of the target population (49).

Information and communication technologies (ICTs) have great potential to increase PA levels and reduce SB levels, at least in the short term. Training protocols delivered via a website, similar to mobile apps, appear capable of improving not only physical fitness but also PA levels, at least in the short term (50). Wearable activity trackers and apps, used alone or in conjunction with websites, can increase step count and average daily moderate PA outdoors (50). A scoping review reported that users are placing greater importance to the “playfulness” on the personalized features, in addition to basic functional requirements, and the main factors related to technological features, including PA tracking, setting PA goals, and exercise personalization, are particularly useful to PA application developers or PA intervention designers (51). Another study demonstrates the potential of gamified digital interventions to promote and maintain PA, offering an alternative to face-to-face programs (52). In parallel, a study has shown that social media use and interactive communication can increase PA, and that posting information about PA on these platforms has a positive impact on users across different population groups (adolescents, students, adults) (53). For example, the PA levels of adolescents (boys and girls) are similar to those of their close friends, both on social media and in real life (53). Numerous studies have established a link between socioeconomic status (SES) and disparities in health behaviors, both within and between countries (54, 55). A meta-analysis of digital interventions targeting PA based on SES found no effect in subgroups defined as having low SES, but a statistically significant effect in participants with high SES (56).

Digital and human interaction is directly linked to daily PA adherence (49). A study has revealed high rates of retention and adherence to the PA program using Fitbit (wearable PA tracker), and is largely related to interactive research staff (via email, during the pre-screening telephone interview and during in-person visits), participant motivation (PA tracking and receipt of personalized feedback based on individual goals) and incentives (49).

Although new technological tools have proven effective, alone or in combination, in children and adults, their effectiveness in increasing PA levels in the elderly remains uncertain (50). A thematic analysis identified the main obstacles to the use of digital tools in primary care for providing PA advice (57). Key factors influencing access to PA include the limited number of digital tools available to encourage or facilitate the provision of such advice, time constraints, the effectiveness, simplicity, and ease of use of digital tools, their integration with existing systems, lack of access to these tools, and lack of technical support for their use (57).

We found that digital communication interventions using computer prompt, mobile and wearable technologies can be effective in reducing SB (21, 22, 24, 32–35). A meta-analysis revealed that sedentary reduction interventions using computer, mobile and wearable technologies in the intervention group resulted in an average reduction of 41 min per day for interventions targeting sedentary time. Moreover, interventions targeting SB in the workplace showed an average decrease of 40 min per workday and interventions targeting overall daily SB showed an average decrease of 45 min per day at the endpoint of follow-up (58).

In our overview of reviews, the BCTs most frequently associated with reducing SB across all interventions were multi-component interventions (computer, mobile, educational materials, scheduled reminders, and wearable devices) + “social support (via social media)”, “self-monitoring”, and “goal setting” (21, 22, 24, 32–35). Some BCTs (especially used in multi-component ones) are better suited to certain delivery methods and that those identified in the technology-based interventions could be usefully integrated into future technology-based interventions aimed at reducing SB. SB differ considerably between the workplace and everyday life in general. Gardner et al. (59) suggested that SB at work are easier to plan and routinize than SB outside of work, which occur in less predictable and structured contexts. This underscores the need to select interventions based on what is most appropriate and feasible in the specific context (59).

A qualitative study explored employees’ perspectives on the factors influencing their ability to reduce SB while working remotely (60). The results show that employees want the autonomy to choose when and how to reduce their sedentary time (60). They want companies to support them and integrate a social dimension into their interventions (60). A lack of confidence felt by employees when working from home discourages them from leaving their office (60). It is essential to inform those who are working from home about the harmful health consequences of prolonged SB and to encourage them by providing adequate rewards (60). A multi-component approach could solve this problem by including the following dimensions “social support”, “self-monitoring”, “goal setting”, and “rewards”.

In our overview of reviews, digital interventions significantly improved sleep quality in adolescents, college students, young and older adults (46–48). Sub-groups analysis in young adults revealed that digital CBT-I had a greater impact on improving sleep quality than other digital sleep interventions, and that therapist-guided interventions were more effective than non-therapist-guided ones (47). These results suggest that therapist-guided digital CBT-I is a particularly effective approach for managing sleep disorders. For older adults, the included review describes the two main approaches used to improve sleep quality: CBT-I delivered through mHealth technologies and the promotion of healthier behaviors, such as PA (using actigraphy) and other lifestyle habits that positively impact sleep quality (46). The greater effectiveness of sleep interventions in adolescents and young adults could be explained by the integration of highly interactive and personalized components, such as sleep diaries, digital cognitive behavioral therapy exercises, and automated feedback systems, which facilitate self-monitoring and self-regulation (47, 48). Conversely, the weaker and more heterogeneous effects observed in older adults could be due to the predominance of less interactive interventions, a lower level of personalization, and difficulties in technology adoption within this population (46).

Digital CBT-I is well-positioned to be the first-line intervention for insomnia and sleep disorders (61). The following five themes were identified as relevant to CBT-I engagement: interpersonal digital components, type and extent of information, user sense of autonomy, application functionality, and importance of personalized content (61). Several barriers were cited in the literature, including the lack of self-efficacy, the knowledge, the desire for additional human support, the perception that digital CBT-I did not take into account clinical complexities, and factors that hindered the implementation of key treatment recommendations (61, 62).

The number of studies on sleep quality is small and the sample sizes identified in the literature are limited (46–48), making the conclusions uncertain. However, the consistency of the results in the selected publications is promising and encourages further studies evaluating the effectiveness of interventions aimed at improving sleep quality, particularly in older people, through mHealth and other digital communication tools.

Our results confirmed the positive impact of apps and mhealth interventions in improving nutritional behaviors. Overall, feedback and monitoring, self-monitoring, goals and planning, knowledge shaping, prompts and cues, and rewards and incentives emerged as the dominant BCT clusters underpinning the effectiveness of digital dietary interventions (36, 37, 42, 43). The more pronounced effects observed in adolescents and young adults could be explained by the integration of highly personalized and interactive features, such as tailored dietary feedback, food diaries, self-monitoring, reminders, gamification, challenges, and rewards, which enhance engagement, motivation, and self-regulation (37, 43). Similarly, interventions combining nutrition education, personalized feedback, and behavioral monitoring have been associated with improvements in eating behaviors and adherence to recommendations (36). In contrast, more modest effects can be observed when interventions rely primarily on the transmission of information with limited personalization or interactive support, highlighting the importance of engagement-driven behavior change strategies to sustain dietary improvements (41, 43).

Several factors can influence the adoption of digital dietary tools. Among them, children and adolescents from disadvantaged socioeconomic backgrounds often face obstacles to adopting healthier eating habits due to limited access to essential resources, increasing their risk of excessive weight gain, obesity, and malnutrition (63). Other factors include access to Wi-Fi, having a mobile phone, tablet, or computer, and understanding how to access and use mobile platforms and applications (64). In addition, An individual's language preferences may impact their preferred method of obtaining health information (face-to-face or technology) (65).

This overview of reviews has strengths and limitations. The main strength was the approach used to search, screen, and synthesize recent literature. All studies were double screened, and data were extracted by two trained researchers, in an attempt to minimize bias and errors. The main limitation was the lack of included studies that focused on healthy behavior including all dimensions (diet, PA, SB, and sleep quality). Even studies targeting only two of the four dimensions (PA and SB) do not examine the inter-relationships. As few studies reported on sleep quality, we could not assess potential effects of digital interventions and their direct effect on BCTs and on participants’ health.

## 5 Conclusion

Digital communication interventions have been used to promote healthy eating habits, physical activity, and sleep quality among adolescents, young adults, and older adults, with mixed results in terms of adherence and effectiveness. However, maintaining long-term engagement and impact remains a major challenge directly related to sustaining behavioral change, even though many interventions lose their effectiveness after only a few weeks.
