# Supplementary material for: Digital communication tools used to promote healthy lifestyles: an overview of reviews from the BRIDGE project
Source: Front Digit Health. 2026 Jul 30;8:1805124. doi: 10.3389/fdgth.2026.1805124 (PMC13469974; doi:10.3389/fdgth.2026.1805124)
Supplement: Supplementary file 1 [file Datasheet1.pdf]

Supplementary Table 1. Search input.

Database	Search String
PubMed	("digital health" OR "mobile application*" OR "smartphone app*" OR wearable* OR "electronic device*" OR "social media" OR website* OR television OR "text messaging" OR "ChatGPT-delivered intervention*") AND ("sedentary behavior" OR "sitting time" OR "physical activity" OR "diet" OR "dietary behavior" OR " healthy eating" OR " nutrition" OR "sleep quality") AND ("behavior change technique*" OR personalization OR interactivity) AND ("systematic review" OR "meta-analysis")
ScienceDirect	("digital intervention") AND ("sedentary behavior" OR "physical activity" OR "dietary behavior" OR "sleep quality") AND ("behavior change technique") AND ("systematic review" OR "meta-analysis")
Cochrane	("digital health" OR "mobile application*" OR "smartphone app*" OR wearable* OR "electronic device*" OR "social media" OR website* OR television OR "text messaging" OR "ChatGPT-delivered intervention*") AND ("sedentary behavior" OR "sitting time" OR "physical activity" OR "diet" OR "dietary behavior" OR " healthy eating" OR " nutrition" OR "sleep quality") AND ("behavior change technique*" OR personalization OR interactivity) AND ("systematic review" OR "meta-analysis")

Supplementary Table 2. Summary of digital communication tools used to promote healthy behaviors

Study/ year	Behavior targeted	Digital communication tools	Degree of personalization	Interactivity	BCT clusters	Main findings
Alley et al., (2024) (30)	Increase PA	Wearable or activity trackers, websites, mobile apps, SMS, e- and/or m-health, and computer-delivered interventions	Moderate to high (personalized feedback based on activity data)	High (real-time feedback, dashboards, goal-setting features)	Self and goal monitoring, feedback on behavior, and reminders	Digital interventions for PA had a moderately significant effect on increasing step count and a small but significant effect on increasing moderate to vigorous intensity PA (MVPA) and total PA.<br>Results did not differ according to the type of measurement (self-reported or via activity tracker)
Beck Silva et al., (2024) (36)	Healthy eating	Computer-based interventions (websites, online educational programs, and CD-ROM-based programs delivered in school settings)	Low to moderate (most interventions provided standardized educational content)	Moderate (interventions commonly included quizzes, educational modules, self-assessment activities, interactive learning exercises, and online engagement components)	Knowledge shaping, goals and planning, feedback and monitoring, self-regulation, and prompts/cues	Computer-based interventions significantly reduced fat consumption among adolescents, but had no significant effect on fruit and vegetable consumption
Bi et al., (2024)(14)	PA	Social media, smart text messaging,	Low to moderate (some interventions included	Moderate to high (self-monitoring, activity tracking,	Goal setting, action planning, feedback and monitoring, social	Digital health interventions significantly increased

		mobile digital applications, smart wearable technology devices (such as smartwatches and pedometers), or a combination of social media and smartphone apps, or a combination of social media and wearable technology devices.	individualized feedback, self-monitoring, tailored goals)	automated feedback, reminders, online engagement, and user interaction with apps or wearable devices).	support, and associations/prompts and cues (reminders and notifications)	the number of daily steps among students, but did not significantly improve MVPA, light-intensity physical activity (LIPA), or time spent sitting.
Buja et al., (2024) (15)	PA	Social media platforms (Facebook groups, specialized platforms, and WeChat), SMS/text messaging, and smartphone-based communication.	Low to moderate (most interventions convey standardized messages; some adapt the content according to the level of activity, objectives or participation of the group).	Moderate to high (any interventions included peer interaction, social groups, challenges, feedback, and online discussions. The social group components appeared to improve effectiveness).	Goals and planning, feedback and monitoring, social support, comparison of behavior, prompts/cues, reward, and incentives	Interventions combining digital communication with social-group engagement were generally more successful. Considerable heterogeneity existed across studies.
Chatterjee et al., (2021)(8)	Healthy lifestyle (physical activity, healthy habits, and proper	smartphone app–based intervention (health monitoring and personalized recommendation generation) and web-	High (many interventions included personalized recommendations, tailored feedback, goal assessment,	Moderate to high (interventions typically involved self-monitoring, self-reporting, feedback generation, goal	Goals and planning (goal setting); feedback and monitoring (self-monitoring, personalized feedback); shaping knowledge/persuasion (persuasive strategies,	Effective interventions can promote behavioral change and improve PA, eating habits, and weight management. Key factors contributing to effectiveness include

	dietary patterns)	based intervention (web-based monitoring and self-management or self-reporting program)	health risk prediction, and individualized follow-up).	setting, engagement features, coaching, and two-way communication with users).	education); self-belief and motivation (self-motivation, empowerment); social/behavioral regulation (engagement, coaching, adherence support)	self-monitoring, self-motivation, goal setting, personalized feedback, participant engagement, psychological support, persuasion, digital skills, efficiency, and credibility.
Chen et al., (2025) (34)	SB	Smartphones, mobile apps, wearable activity monitors (e.g., accelerometers), mobile prompts/reminders, mobile monitoring devices.	Moderate (several interventions used individualized goals, tailored feedback, and monitoring based on participants' activity data, although personalization levels varied across studies)	Moderate to high (interventions commonly included self-monitoring, feedback, reminders/prompts, goal tracking, and participant engagement with mobile devices)	Goal setting, problem solving, action planning, review behavior goals, feedback and monitoring, and self-regulation	Interventions carried out via smartphones and wearable monitoring devices, combined with behavior change techniques, have proven effective in reducing sitting time among older people
Curtin et al., (2025) (42)	Healthy and sustainable diet behaviors	Researcher-developed apps: mobile diet apps, dietary apps, dietary monitoring apps, self-tracking tools, in-app coaching	High (feedback and monitoring)	High (goal setting, and feedback loops)	Feedback, monitoring, and goals and planning	The apps generally resulted in favorable changes in food consumption (mainly an increase in fruit and vegetable consumption), with a peak around 12 weeks after the start of app use
D'Amore et al., (2022)(16)	PA	Smart technologies (mobile applications, wearable activity trackers, telehealth	Moderate to high (many interventions incorporated individualized	High (frequent use of self-monitoring, real-time feedback, videoconferencing,	Goals and planning, feedback and monitoring, social support, instruction on	Smart technology-based interventions have proven as effective as face-to-face

		platforms, videoconferencing systems, web-based exercise programs, exergames (interactive video games), activity-monitoring devices, and remote coaching technologies)	exercise prescriptions, tailored activity goals, remote monitoring, adaptive feedback, and coaching based on participant progress and health status)	remote coaching, progress tracking, interactive exercise sessions, communication with health professionals, and technology-assisted engagement)	how to perform the behavior, behavioral practice/rehearsal, self-monitoring, and prompts/cues	interventions for PA among older adults, justifying their use as an alternative or complement to traditional programs. Interventions combining remote support and smart technologies have shown promising results in terms of maintaining participation and adherence
Daniels et al., (2025) (31)	Increase PA	Application, websites, wearable device, website + wearable device, and application + wearable device.	High (step goals, reminders, and user-centered design)	High (real-time feedback)	Self-monitoring and monitoring (planning, coaching and social support)	A potential positive effect of mobile health on increasing PA in older adults.<br>Interventions varied considerably, ranging from simple mobile health applications to complex, multi-component strategies.<br>Variability in effectiveness, sample size, methodology, study duration, and adoption across studies presents a complex picture
Goodyear et al., (2021)(17)	PA and dietary behaviors	Social media platforms (Facebook, Instagram, Twitter,	Low to moderate (standardized content to groups	High (peer interaction, commenting,	Social support, social comparison, goals and planning, feedback and	Social media interventions have shown potential to

		YouTube, WhatsApp, Reddit, WeChat, online social networking sites, discussion groups, blogs, and social media communities)	through social media)	sharing, discussion forums, social networking, challenges, and group participation)	monitoring, rewards and incentives, prompts/cues	improve PA and eating behaviors by increasing social support, engagement, and motivation, but results are mixed and heterogeneous. The effects are generally more pronounced when social media is combined with other behavior change strategies
Grotto et al., (2024) (46)	Sleep quality	Digital CBT-I technologies (tablets and smartphones), mHealth apps.	Moderate (CBT-I mobile app, and mobile health interventions)	Moderate to high (cognitive behavioral restructuring)	Self-monitoring, goal setting, reminders, feedback, and sleep diary	mHealth interventions were effective in improving the subjective sleep quality of older adults
He et al., (2021)(18)	Increase PA	Smartphone-based intervention technologies (mHealth, app, SMS text messaging, wearable-linked smartphone apps, and digital coaching platforms)	Moderate (some interventions included tailored goals, feedback based on activity data, individualized step targets, or adaptive recommendations, but many were standardized app features)	Moderate to high (interventions commonly included self-monitoring, feedback, reminders, goal tracking, and app-based engagement features)	Goals and planning, feedback and monitoring, prompts/cues, shaping knowledge, self-belief, reward, and reinforcement	Smartphone-based interventions significantly improve PA (particularly step count and MVPA) in children and adolescents, with even more pronounced effects when self-monitoring, goal setting, and feedback are included. However, the long-term sustainability of these effects has not been systematically assessed

He et al., (2024) (33)	Reduction of SB and prolonged sitting time	Wearable devices solely, wearable devices + online interventions (internet platforms), and wearable devices + offline interventions (face-to-face seminars and courses)	Moderate (most wearable interventions provided individualized monitoring, personalized activity goals, tailored sedentary-time alerts, and feedback based on users' activity patterns)	High (self-monitoring, automated feedback, real-time prompts to interrupt sitting, progress tracking, reminders, and integration with mobile applications)	Feedback and monitoring, self-monitoring of behavior, prompts/cues, goals and planning, behavioral regulation, and action planning	Combining wearable devices + online tools (personalized reminders and feedback) represented the best intervention strategy for reducing sedentary time in healthy adults. However, no significant difference was observed between the groups regarding the reduction of prolonged sitting
Laranjo et al., (2021) (19)	PA	Smartphone applications (mobile apps) and wearable activity trackers (e.g., Fitbit and similar devices)	Moderate to high (most interventions included individualized goals, tailored feedback, self-monitoring based on personal activity data, and adaptive progress tracking)	High (self-monitoring, real-time feedback, goal setting, progress visualization, reminders, notifications, rewards, and social interaction features)	Goals and planning, feedback and monitoring, self-monitoring, rewards and incentives, social support, prompts/cues, and behavioral regulation	Digital interventions using smartphone apps and activity trackers are effective tools for increasing PA, with more pronounced effects when multiple BCTs and personalized feedback are integrated
Leppe-Zamora et al., (2025) (32)	Reduce SB and increase active breaks in sitting time	Computer-based prompts (+ educational information + phone calls or text messages), computer prompt + wrist-worn devices, computer	Low (prompts used for all users)	Low (automated prompts)	Reminders using computer prompt interventions	Computer prompt interventions effectively reduce SB in the workplace

		prompt + an exercise video, computer prompts + sit-stand workstation				
Lu et al., (2025) (47)	Sleep quality	Mobile apps, web-based sleep programs, videoconferencing, cognitive behavioral therapy for insomnia (CBT-I) apps, virtual reality (meditation)	High (tailored CBT-I, and sleep education programs delivered via web-based platforms or mobile apps)	Moderate to high (digital modules, cognitive behavioral restructuring)	Goal setting<br>Self-monitoring<br>Text message<br>Sleep diary	Digital sleep interventions significantly improved sleep quality
Melo et al., (2025)(43)	Dietary behavior	Digital dietary interventions (mobile applications, websites, web-based platforms, online programs, social media components, text messaging systems, and digital educational tools)	Moderate to high (personalized dietary feedback, tailored recommendations, individualized goals, adaptive content, and self-monitoring functions)	High (dietary self-monitoring, progress tracking, gamification, quizzes, challenges, rewards, reminders, peer interaction, and automated feedback)	Goals and planning, personalized feedback, self-monitoring of behavior, knowledge shaping, rewards and incentives, prompts/cues, social support, and behavioral regulation	Digital interventions related to nutrition have generally improved adolescents' eating habits. Interventions combining personalized feedback and gamification have achieved the highest engagement and adherence rates
Mersha et al., (2024) (20)	PA, dietary behaviors, weight management, smoking cessation.	Internet-based group behavioral interventions (websites, Facebook, mobile/computer applications, Twitter, WeChat, Whatsapp, online group sessions, and discussion forums)	Low to moderate (group-based support. Some studies incorporated goal setting, or tailored content)	High (interaction through online group discussions)	Social support, goals and planning, feedback and monitoring, problem solving, behavioral practice/rehearsal, self-monitoring, and social comparison	Internet-based group behavioral interventions have generally proven effective (in the short term of roughly up to 6 months) in supporting lifestyle-related behavior changes, including increasing PA, improving eating habits,

						achieving weight-related goals, and strengthening participant engagement
Paramastri et al., (2020) (44)	Dietary intake, and PA.	mHealth applications (smartphone apps) used for diet tracking, nutrition education, health coaching, reminders, self-monitoring, and feedback	Moderate to high (many apps included individualized dietary monitoring, tailored feedback, personalized goals, reminders, and coaching features)	High (users actively recorded dietary intake, monitored progress, received feedback, interacted with coaching functions, and used reminder systems)	Feedback and monitoring, goals and planning, shaping knowledge, prompts/cues, social support/coaching, self-belief	Mobile applications can improve nutrition-related behaviors through dietary tracking, feedback, reminders, coaching, and nutrition education. Most of the included studies reported positive effects on dietary outcomes and the adoption of healthier behaviors, although the data remain limited and heterogeneous
Parés-Salomón et al., (2024) (35)	Reduce SB	Information delivery (digital media "text, videos"), digital log (mobile phone diary for self-monitoring), wearables (activity trackers), scheduled prompts, automated tailored feedback (online forums, social influences)	Moderate to high (diaries, activity trackers, and online forums)	High (wearables, app notifications, computer, feedback)	Goal setting<br>Self-monitoring<br>SMS interventions<br>Social media	The most effective interventions were multi-component and included a wide variety of digital features, with the dissemination of information (text messages, electronic newsletters, websites, and videos) and educational materials followed by scheduled prompts
Peng et al., (2022) (21)	PA and SB	E-health interventions (smartphone	Moderate (goal setting, tailored	High (self-monitoring,	Goals and planning, feedback and	E-health interventions have a significant impact

		apps, social media platforms, wearable activity trackers, and online interventions).	feedback, personalized activity monitoring, and adaptive recommendations)	activity tracking, feedback, reminders, social interaction, online coaching, challenges, and digital engagement through apps and web platforms)	monitoring, self-monitoring of behavior, social support, prompts/cues, behavioral regulation, and rewards/incentives	on increasing PA, including TPA, MVPA and the number of steps at post-intervention. However, the maintenance of this improvement during follow-up and the effect of the interventions on reducing SB remain to be studied
Petkovic et al., (2021) (29)	PA	Interactive social media (Facebook groups, WeChat, Twitter, WhatsApp, and Google Hangouts. online communities, discussion forums, social networking platforms)	Moderate (some tailoring through individualized feedback, peer interactions, and self-monitoring data)	High (active user participation through posting, commenting, peer interaction, discussion groups, social support, sharing progress, and receiving feedback)	Social support, goals and planning, feedback and monitoring, social comparison, reward and reinforcement, shaping knowledge	Interactive interventions on social media may increase PA through social support, engagement, self-monitoring and feedback mechanisms, but the level of evidence is low and the effects remain uncertain
Salamanca-Sanabria et al., (2025) (48)	Sleep behavior	Digital health interventions (DHIs) (mobile apps, web-based platforms, online sleep programs, digital cognitive behavioral therapy (d-CBT), wearable-supported systems, SMS/email support, and other	Moderate to high (tailored sleep recommendations, individualized feedback, adaptive goal setting, personalized sleep monitoring, and behavior-specific guidance)	Moderate to high. (self-monitoring, automated feedback, educational modules, reminders, digital coaching, interactive exercises, sleep	Feedback and monitoring, goals and planning, self-monitoring of behavior, knowledge shaping, prompts/cues, behavioral regulation, problem solving, and action planning	Trials of d-CBT have shown significant improvement in insomnia among adolescents. Educational, sleep hygiene, and holistic promotion interventions have yielded more variable results, with less

		internet-based technologies)		diaries, and progress tracking)		improvement in sleep after treatment
Scarry et al., (2022) (45)	Diet quality improvement, and healthy eating behaviors	Mobile applications (smartphone apps), mHealth technologies, web-linked apps, wearable-connected platforms, dietary assessment and tracking tools	Moderate to high (many interventions incorporated personalized dietary feedback, individualized recommendations, goal setting, tailored messages, and self-monitoring feature)	High (users actively engaged in dietary logging, self-monitoring, progress tracking, receiving feedback, goal review, and interaction with app-based functions)	Feedback and monitoring, goals and planning, shaping knowledge, prompts/cues, self-belief, behavioral regulation	Mobile app-based interventions have improved diet quality and healthy eating behaviors, particularly when they include self-monitoring, personalized feedback, goal setting, and education. Overall, mhealth has shown promise in promoting dietary behavior change, although the evidence is limited by study heterogeneity and inconsistency in diet quality measures
Schaafsma et al., (2024) (37)	Dietary intake	Smartphone app-based interventions (mobile applications).	Moderate to high (personalized feedback, self-monitoring functions, messaging from a healthcare provider, and adaptive progress tracking)	High (self-monitoring, interactive food logging, gamification, quizzes, rewards, push notifications, progress dashboards, and user engagement features)	Goals and planning, feedback and monitoring, self-monitoring of behavior, rewards and incentives, prompts/cues, knowledge shaping, and behavioral regulation	Smartphone app-based interventions have generally improved adolescents' eating behaviors and nutrition-related outcomes, particularly through self-monitoring, goal setting, feedback, and gamification, although evidence of their long-term effectiveness remains limited

Shaluhiyah et al., (2025) (38)	PA and diet	DHIs (messaging platforms, social media platforms, mobile apps, gamification and interactive media, wearables and monitoring, video conferencing tools, web-based tools)	Moderate to high (tailored messages, personalized feedback, individualized goal setting, adaptive recommendations, risk profiling, and behavior-specific coaching)	High (self-monitoring, real-time feedback, bidirectional communication, online coaching, reminders, peer support, gamification, social networking, progress tracking, and virtual counseling)	Goals and planning, feedback and monitoring, self-monitoring of behavior, social support, knowledge shaping, prompts/cues, behavioral regulation, rewards and incentives, and problem solving	Messaging platforms and mobile applications have proven to be the most frequently used and effective digital tools for preventing NCD. Multimodal interventions and platform-to-outcome fit appear essential for maximizing the effectiveness of digital health programs promoting healthy behaviors
Sina et al., (2022) (39)	Diet	Social media platforms (Instagram, YouTube, Facebook, and WhatsApp), influencer content, peer-generated content, food marketing and advertising delivered through social media	Low to moderate (most exposures were not personalized health interventions; however, algorithmically driven content, targeted advertising, influencer marketing, and personalized feeds created indirect personalization)	High (users actively engaged by liking, sharing, commenting, following influencers, viewing content from their peers, and participating in online communities)	Social support, social comparison, behavior modeling/demonstration, persuasion, environmental restructuring (digital food environment) and rewards/incentives (through social approval and engagement indicators)	Social media may significantly influence the food preferences, food choices, and eating behaviors of children and adolescents. The study identified several mechanisms: (1) social norms and peer influence; (2) the credibility and identification of influencers; (3) food marketing and advertising; (4) visual cues of food triggering physiological responses

						(appetite, cravings); and (5) reinforcement through engagement and social validation
Talens et al., (2025) (40)	Dietary behaviors, and PA	Mobile applications, web-based programs, serious games/ gamified interventions, eHealth platforms, educational websites, digital learning modules, and other interactive digital tools.	Low to moderate (most interventions provided standardized content, although some included individualized feedback, self-monitoring, tailored goals, or family-based components).	Moderate to high (many interventions required active engagement through games, quizzes, challenges, progress tracking, self-monitoring, interactive learning activities, and user participation)	Shaping knowledge (nutrition education), goals and planning (behavioral goals), feedback and monitoring (tracking and self-monitoring), reward and reinforcement (gamification, points, rewards), social support (family- or school-based participation in some studies), behavioral practice (repeated healthy-choice activities)	Digital interventions have proven effective in improving children's and adolescents' eating habits (especially fruit intake and healthy food choices) and nutritional knowledge, particularly through game-based approaches, but have shown limited and inconsistent effects on PA
Tong et al., (2021) (28)	PA and diet	Mobile applications, SMS/text messaging, wearable device linked mobile systems, smartphone-based coaching systems, mobile monitoring platforms, and other mHealth technologies	High (interventions delivered personalized recommendations, tailored feedback, adaptive goal setting, context-aware messaging, and individualized coaching)	High (most interventions incorporated self-monitoring, bidirectional feedback, progress tracking, tailored messaging, reminders, and adaptive user engagement)	Feedback and monitoring, goals and planning, shaping knowledge, prompts/cues, behavioral regulation, self-belief, and social support	Personalized mobile technologies have led to modest but significant improvements in health behaviors, particularly PA. Interventions combining multiple personalization strategies have generally proven more effective, while results regarding dietary improvements

						were positive but less consistent
Tong et al., 2024 (22)	Increase PA and reduce SB	Mobile apps, SMS, wearables, fitness trackers.	Low (limited tailoring and adherence)	Moderate (app notifications, SMS interventions, social media)	Goal setting, self-monitoring, SMS interventions, and social media	In the MENA region, mobile interventions for PA and SB are in their early stages, with preliminary evidence indicating their effectiveness
Wang et al., (2025) (24)	PA and SB	Gamified digital interventions (active video games, mobile applications, web-based platforms, wearable-connected games, virtual reality environments, and digital programs)	Low to moderate (individualized goals, adaptive challenges, personalized feedback)	Very High (active participation and engagement through challenges, rewards, competitions, progress tracking, peer interaction, feedback, and game-based tasks)	Rewards and incentives, feedback and monitoring, goals and planning, social support, social comparison, behavioral practice/rehearsal, prompts/cues, and identity enhancement	Gamification interventions significantly increased MVPA. However, no significant improvements were observed in SB, VPA, MPA, LPA, or daily step count. This effectiveness is considerably modulated by theoretical paradigms, game elements, and the duration of the intervention
Wang et al., (2025)(25)	PA, exercise habits, and SB reduction	Chatbot-based exercise interventions (conversational agents integrated into mobile apps, messaging platforms, websites, and digital health systems)	High (tailored exercise recommendations, personalized goal setting, adaptive feedback, individualized coaching, and	Very High (continuous two-way communication, real-life situation feedback, conversational coaching,	Goals and planning, feedback and monitoring, self-monitoring of behavior, prompts/cues, social support (virtual coaching), problem solving, behavioral	Chatbot-based interventions significantly improved PA and exercise habits. Their effectiveness appears to rely on personalized guidance, real-time feedback, and

			responses based on users' behavior, preferences, and progress)	reminders, progress monitoring, motivational messaging, and interactive problem-solving)	regulation, and action planning	continuous interactive support. However, it did not exert significant impact on exercise habits and SB
Wang et al., (2024) (23)	Increase PA and fitness	Mobile health apps (commercial apps, research apps)	High (gamification, age-tailored challenges)	Extremely high (rewards, games, interactive missions)	Gamification, self and goal monitoring, rewards, social support, shaping knowledge	mHealth app-based interventions increased total PA and reduced SB among children and adolescents but had no significant effect on MVPA.<br>Gamification is highly effective for youth
Watson-Mackie et al., (2024) (26)	PA	Technology-supported PA interventions (mobile applications, wearable activity trackers, online programs, web-based interventions, and social media-supported technologies)	Low to moderate (individualized goals, adaptive challenges, personalized feedback, or user-selected activities)	High (active engagement through gamification, self-monitoring, real-time feedback, interactive challenges, virtual environments, social networking features, and peer competition /collaboration)	Goals and planning, feedback and monitoring, social support, rewards and incentives, behavioral practice/rehearsal, and prompts and cues	The results did not indicate that technology-supported PA is an effective method for increasing PA among young women, although, when the results are broken down by age group, technology-supported PA may have a greater impact on PA engagement among young adults than among adolescents

Xu et al., (2022) (27)	PA	Gamified mobile apps, wearable-linked mHealth platforms.	Low to moderate (most interventions were centered on gamification rather than intensive tailoring. Some studies included personalized goals, adaptive feedback, and individualized activity targets)	Very high (users actively interacted with challenges, competitions, rewards, leaderboards, badges, points, progress tracking, and social engagement features)	Goals and planning, feedback and monitoring, reward and reinforcement, social support, social comparison, behavioral practice/repetition, and self-belief	Mobile health interventions based on gamification have improved participation and engagement in PA, particularly through rewards, competition, and social interaction, although evidence of their long-term effectiveness remains limited
Zarnowiecki et al., (2020) (41)	Nutrition	Websites, smartphone apps (meal planner, shopping list), mobile applications, web-based nutrition programs, SMS or email-supported digital interventions	Moderate to high (tailored feedback, personalized content, and customized recommendations)	Moderate (combined educational information with interactive features)	Knowledge shaping, feedback and monitoring, goals and planning, social support, prompts/cues, and self-efficacy enhancement	Digital nutrition interventions for parents have generally improved infant nutrition, parental knowledge, and parents' sense of self-efficacy. These interventions have proven most promising when they incorporate interactive features, tailored feedback, and personalization, rather than simply disseminating information

BCTs: behavior change techniques, CBT-I: cognitive behavioral therapy for insomnia, LPA: light-intensity physical activity, MPA: moderate physical activity, MVPA: moderate-to-vigorous physical activity, PA: physical activity, VPA: vigorous physical activity.
